# Mixed invasive fungal infections among COVID-19 patients

**DOI:** 10.18502/cmm.7.4.8407

**Published:** 2021-12

**Authors:** Vanya Singh, Amber Prasad, Prasan Kumar Panda, Manjunath Totaganti, Amit Kumar Tyagi, Abhinav Thaduri, Shalinee Rao, Mukesh Bairwa, Ashok Kumar Singh

**Affiliations:** 1 Department of Microbiology, All India Institute of Medical Sciences, Rishikesh, Uttarakhand, India; 2 Department of Internal Medicine, All India Institute of Medical Sciences, Rishikesh, Uttarakhand, India; 3 Department of ENT, All India Institute of Medical Sciences, Rishikesh, Uttarakhand, India; 4 Department of Pathology, All India Institute of Medical Sciences, Rishikesh, Uttarakhand, India

**Keywords:** Aspergillosis, Coronavirus disease, Invasive fungal disease, Mucormycosis

## Abstract

**Background and Purpose:**

The healthcare system in India collapsed during the second wave of the COVID-19 pandemic. A fungal epidemic was announced amid the pandemic with several cases of COVID-associated mucormycosis and pulmonary aspergillosis being reported. However, there is limited data regarding mixed fungal infections in COVID-19 patients. Therefore, we present a series of ten consecutive COVID-19 patients with mixed invasive fungal infections (MIFIs).

**Materials and Methods:**

Among COVID-19 patients hospitalized in May 2021 at a tertiary care center in North India, 10 cases of microbiologically confirmed COVID-19-associated mucormycosis-aspergillosis (CAMA) were evaluated.

**Results:**

All patients had diabetes and the majority of them were infected with severe COVID-19 pneumonia (6/10, 60%) either on admission or in the past month while two were each of moderate (20%) and mild (20%) categories of COVID-19; and were treated with steroid and cocktail therapy. The patients were managed with amphotericin-B along with surgical intervention. In total, 70% of all CAMA patients (*Rhizopus arrhizus* with *Aspergillus flavus* in seven and *Aspergillus fumigatus* complex in three patients) survived.

**Conclusion:**

The study findings reflected the critical importance of a high index of clinical suspicion and accurate microbiological diagnosis in managing invasive dual molds and better understanding of the risk and progression of MIFIs among COVID-19 patients. Careful scrutiny and identification of MIFIs play a key role in the implementation of effective management strategies.

## Introduction

Over the past one and half years, the severe acute respiratory syndrome coronavirus-2 (SARS-CoV-2) has presented with a myriad of manifestations and complications [ 1 , 2 ]. While the world managed to recover from the first wave of COVID-19, the second wave has put the world at its wit's end, with the SARS-CoV-2 virus bringing up new surprises and tricks. The second wave has especially hit hard on India and increased the incidence of invasive fungal infections dramatically.

The usually rare acute invasive fungal rhinosinusitis cases have surged in post-COVID patients [ 3 - 5 ]. Since there is a lack of population-based studies in the literature to estimate the baseline incidence/prevalence rate of mucormycosis in India, it is difficult to state that the overall incidence rate of COVID-19-associated pulmonary mucormycosis (CAPM) has increased [ 6 ]. COVID-associated pulmonary aspergillosis (CAPA) is another emerging phenomenon which was also reported during the first wave of the COVID pandemic [ 7 ]. However, mixed invasive fungal infections associated with COVID-19 have rarely been reported. 

To the best of our knowledge, it is the first case series of 10 consecutive cases of COVID-associated mucor-aspergillosis (CAMA) with their associated risk factors, clinical presentation, diagnosis, treatment, and outcome.

## Materials and Methods

### Study design and patients

This case series is part of a project entitled “Disease profile of COVID-l9” which includes patients’ follow-up at a tertiary institute in India, approved by the Institutional Ethical Committee in All India Institute of Medical Sciences (AIIMS), Rishikesh, India (CTRI/2020/08/027169). The recorded data included baseline demographics, presenting signs and symptoms, disease characteristics, microbiological and radiological findings, treatments, and mortality outcomes of the study population.

### Inclusion criteria

The inclusion criteria included known cases of COVID infection or positive result of reverse transcriptase-polymerase chain reaction (RT-PCR) test for COVID for those admitted in May 2021 as well as presentation of one or more of the following symptoms: decreased/loss of vision, sinusitis, headache, facial cellulitis, diplopia, proptosis, toothache, loosening of teeth, blackish discoloration over the bridge of nose/palate, prolonged fever, jaw involvement, altered mental status, necrosis of tissue with black crusts. Accordingly, 10 consecutive patients were included in the study whose nasal tissue sample showed both broad aseptate ribbon-like hyphae with perpendicular branching and acute-angle thin hyaline septate hyphae on potassium hydroxide (KOH) mount or whose fungal culture report showed pure growth of both Mucorales and *Aspergillus* species.

### Procedures

The extent and severity of patients’ disease were determined based on their detailed history, comprehensive otorhinolaryngological and neurological examinations, as well as ophthalmologic evaluation. Routine blood investigations were conducted which included complete blood counts, blood sugar and liver function tests, kidney function tests, along with HbA1C, serum procalcitonin, and serum ferritin tests.

Microscopy (direct and/or histopathology) and culture of specimens were the cornerstones of diagnosis. The samples were obtained from scraping or exudate from the nasal cavity and/or paranasal sinuses, hard palatal lesions, sinus material, a biopsy from extracted tooth socket area, and endoscopic collection of debrided tissue/biopsy [ 6 ]. Diagnosis of mixed fungal infections was made if two types of fungal hyphae were visible on direct sample KOH. Diagnosis of Mucorales was made based on a demonstration of broad aseptate ribbon-like hyphae with right-angle branching on 10% KOH preparation of specimens and Sabouraud’s Dextrose Agar culture reports. In contrast, demonstration of acute-angled thin hyaline septate hyphae was suggestive of Aspergillus spp. Lactophenol Cotton Blue (LPCB) was used to stain fungal elements obtained on culture growth [ 8 ]. Histopathological examination was also conducted for the specimens excised during surgery, including sinus, maxilla, and orbital fat. H&E and PAS stains were used to confirm the presence of fungal hyphae.

Radiological investigations, including computerized tomography (CT) and/or magnetic resonance imaging (MRI) of paranasal sinuses, orbit, brain, and thorax were obtained to identify the extent of the patient's disease. The patients were diagnosed as proven, probable, or possible cases of invasive fungal diseases (IFD), based on the European Organisation for Research and Treatment of Cancer and the Mycosis Study Group Education and Research Consortium definitions and ECMM/ISHAM consensus criteria [ 9 ]. Patients with the presence of at least one host factor, a clinical feature, and mycologic evidence were considered to have probable IFD, whereas those with histopathological evidence in addition to the aforementioned factors were considered to have proven IFD. Medical and/or surgical management of the patients was conducted depending on the severity of their disease, based on a mutual decision of a multidisciplinary team [ 10 ]. The treatment outcome was assessed in terms of the success and failure of management. A stable and disease-free patient or stable patient on follow-up presented treatment success, while treatment failure was reflected in the death of the patient due to fungal invasion.

## Results

**Figure 1:**
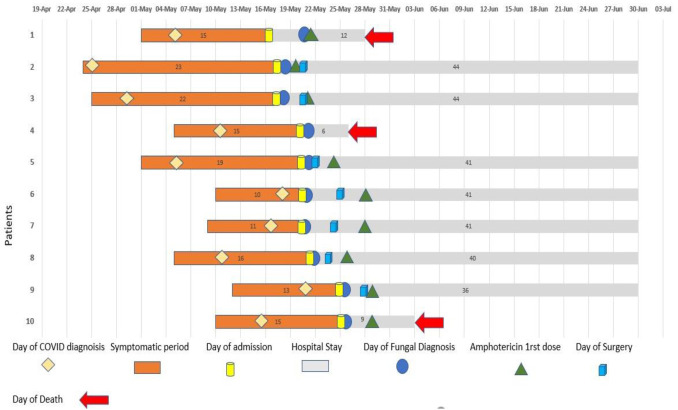
Schematic timeline presentation of 10 COVID-19-associated mucormycosis- aspergillosis cases (from the day of symptom onset to the day of outcome)

The main demographic, clinical characteristics and associated risk factors for fungal infections of 10 patients were documented at hospital admission (Table 1). However, only 6 out of these 10 patients were confirmed cases of COVID-19 before admission. The remaining four cases were never tested for COVID-19 before admission despite having clinical symptoms suggestive of the same and were diagnosed with COVID-19 by reverse transcription polymerase chain reaction (RT-PCR) after admission at our center. Figure 1 represents major events in the course of illness for each case. The participants included six men and four women with a mean age of 49.2±8.8 years (age range of 34-62 years). All patients had a history of COVID-19 within one month (15-23 days) of presentation (Figure 1, Table 1) and were diabetic as well. Moreover, eight patients had uncontrolled diabetes mellitus with poor glycaemic control (HbA1C>7.0), one was pre-diabetic, and one had long-duration diabetes (>7 years) with glycaemic control (Table 1). Furthermore, six patients were previously hospitalized with COVID-associated respiratory distress, and one had received home-based oxygen therapy due to the unavailability of hospital beds at his home place. These seven patients also had documented history of steroid intake for an average of one week.

**Table 1:** Basic characteristics and clinical presentations of 10 COVID-19 associated mucormycosis- aspergillosis patients

Case no.	Age/ Gender	Clinical presentation	Interval between symptoms related to COVID-19 and to fungal infection (in days)	Comorbidities/ immuno-compromised state/ Other risk factors	Initial diagnosis
1	45/ M	Headache, fever, altered sensorium, decrease vision	15	Uncontrolled diabetes mellitus x 3 years; Severe COVID pneumonia; Smoker 1 pack/day for 10 years, H/O Steroid, low dose, > 1 week	Probable Rhino orbital mucormycosis with COVID-19 Probable Aspergillosis
2	46/M	Headache, right nasal obstruction, right periorbital swelling, right eye tear with black nasal discharge	Not tested before admission (COVID symptoms-25 days back)	Uncontrolled diabetes mellitus x 3 years; Severe COVID pneumonia. H/O Steroid intake for 5 -7 days; home-based oxygen therapy, smoker, H/O exposure to saw-dust (carpenter by occupation)	Proven rhino orbital mucormycosis with COVID-19 and proven aspergillosis
3	49/M	Headache, facial swelling, Fever	22	Newly diagnosed uncontrolled diabetes mellitus; Severe COVID pneumonia; H/O Non-invasive ventilation (BiPAP) for 20 days with high dose parenteral steroid > 2 weeks	Proven rhino-orbital mucormycosis and probable aspergillosis
4	62/F	Facial pain, nasal stuffiness, nasal discharge	10	Diabetes mellitus x 10 years; Moderate to severe COVID pneumonia. H/O Oxygen therapy and high steroid intake for 5-7 days	Probable Sinonasal mucormycosis and probable aspergillosis
5	34/F	Headache, swelling, nasal discharge, right eye pain	Not tested before admission (H/O fever with COVID-19 symptoms 20 days back)	Newly diagnosed uncontrolled diabetes mellitus; Mild COVID, Incision and drain in hard palate 10 days before presentation, unsterile dressing, open wound	Proven rhino-orbital mucormycosis with COVID-19 and proven aspergillosis
6	62/M	Eye pain, swelling, facial numbness	Not tested before admission (H/0 fever with cough, 10 days prior to admission)	Diabetes mellitus x 7 years; Moderate COVID, H/O low dose Steroid intake, chronic kidney disease; H/O dialysis; Smoker	Proven rhino-orbital mucormycosis with COVID-19 and proven aspergillosis
7	45/M	Headache, periorbital swelling, vomiting	Not tested before admission (admitted to another center with symptoms suggestive of COVID-19, H/0 fever with cough, 11 days)	Uncontrolled Diabetes mellitus x 4 years, CKD ; Mild COVID, Smoker	Proven rhino-orbital mucormycosis and probable aspergillosis
8	52/M	Headache, facial numbness, swelling, nasal discharge	16	Uncontrolled diabetes mellitus x 4 years; Moderate COVID, Previous hospitalization for COVID-19 for 1 week, H/O high dose steroid intake	Proven rhino-orbital mucormycosis with COVID-19, and proven aspergillosis
9	42/F	Right eye swelling, pain, discharge	5	Diabetes mellitus x 1 years; Severe COVID pneumonia, No other significant history	Proven rhino-orbital mucormycosis with COVID-19 and proven aspergillosis
10	55/F	Fever, cough, breathlessness, swelling of the nose	23	Newly diagnosed diabetes mellitus; Pancytopenia; Severe COVID pneumonia, H/O high dose steroid intake; H/O blood transfusion (5 whole units); Recovered COVID pneumonitis (2/5/21 – 19/5/21)	Probable nasal mucormycosis, probable aspergillosis

Patient No.1 was referred to our hospital and diagnosed as a case of diabetic ketoacidosis with severe COVID-associated pneumonia and sepsis. On days 5 and 9, two consecutive nasal tissue samples were sent to the laboratory for KOH. The results showed acute angle branched thin hyaline septate hyphae, suggestive of *Aspergillus* spp. Subsequently, the patient was started on amphotericin-B injection. The patient’s condition deteriorated; therefore, KOH examination of nasal crusts was repeated five days later to rule out mucormycosis. The results revealed broad aseptate perpendicular branching hyphae and thin hyaline septate hyphae. However, the patient succumbed a day later.

Pre-operative nasal tissue KOH stain of patient no. 2, a 46-year-old carpenter, was suggestive of Mucorales and *Aspergillus*. Although only broad aseptate hyphae were visible in the post-operative sample, the fungal culture of both samples showed growth of R. arrhizus and A. flavus. 

Patients No. 4 and 10 were intubated and managed conservatively. Patients No. 3,7,8, and 9 were diagnosed on the day of admission and were managed with medical therapy aided by endoscopic debridement. Based on the last follow-up, these patients have been recovering well to date.

**Figure 2:**
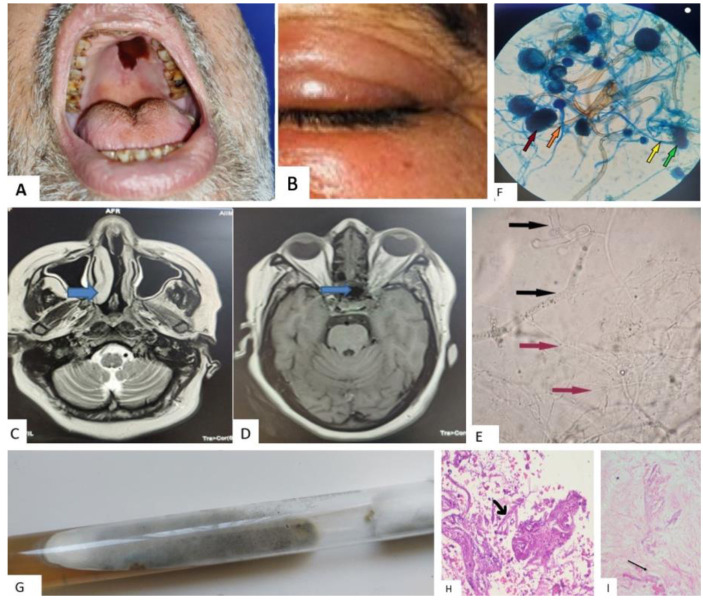
A and B: Patient photographs. Rhino-orbital involvement of COVID-19-associated mucormycosis- aspergillosis patients Nos. 2, 3, 6, 9, showing necrotic scab formation in the hard palate (A), left red-eye with swelling (B) C and D: MRI image of brain involvement in COVID-19-associated mucormycosis- aspergillosis patients. C) T2 weighted MRI image showing hyperintensity in bilateral turbinates and nasal septum, D) T1 weighted MRI showing hyperintensity in bilateral ethmoidal sinuses.**E, F, and G:** Microbiological images demonstrating rhino-orbital-cerebral coronavirus disease-associated mucormycosis and aspergillosis, E) 10 % KOH mount: Black arrows indicating wide-angled broad aseptate hyphae, and Red arrows indicating thin hyaline acute angle septate branched hyphae, F) LPCB mount showing growth of R. arrhizus and A. flavus, dark red arrow indicating sporangium with sporangiospores, orange arrow: sporangiophores of R. arrhizus, green arrow: conidiospore, and yellow arrow: conidiophores of A. flavus, G) SDA culture tube showing growth of Rhizopus spp. and Aspergillus spp. H and I: Histopathology sections (100x, H&E) of nasal tissue, H) Presence of broad, aseptate hyphae and branching at right angles resembling Mucor along with thin septate hyphae (suggestive of Aspergillus spp.), I) Arrow indicating the presence of broad aseptate fungus within the blood vessel (angioinvasion)

Radiological, microbiological, and histopathological diagnosis, treatment, outcome and Laboratory findings of the patients were documented (Table 2, Table 3, Figure 2). Nasal tissue of all patients showed growth of Rhizopus arrhizus with Aspergillus flavus in seven and Aspergillus fumigatus complex in three patients. None of the patients were diagnosed with concurrent candidiasis. Only 70% of patients (7/10) were managed successfully in terms of saving a life

**Table 2:** Radiological and microbiological diagnosis, treatment, and outcome of ten COVID-19 associated mucormycosis- aspergillosis patients

Case no.	Radiological findings	Microbiological findings		Histopathological findings	Management	Surgical	Intra-op findings	Follow-up/ final outcome
		KOH	Culture					
1	Left M & F, B/L E & S -sinusitis	Thin hyaline septate hyphae-1^st^ and 2^nd^ sample, broad aseptate in 3rd sample	*R. arrhizus* and *A. flavus*	Sample not sent	Conservative injection of amphotericin B liposomal 5mg/kg for 7 days and Injection Piptaz	ND	Dead
2	B/L Maxillary(R>L, B/L E & S sinusitis with right orbital extension into extraconal compartment	Broad pauciseptate	*R. arrhizus* & *A. flavus*	Broad aseptate with few thin septate hyphae. Presence of angioinvasion, tissue necrosis, inflammatory cells, and giant cell reaction	Inj. Amphotericin B 1425 mg Cumulative dose, Injection of Augmentin, Metrogyl	Surgical Debridement, Modified Dunker’s approach, Right frontal sinusotomy, septoplasty, left MMA, Right Ant & post ethmoidectomy, left anterior ethmoidectomy, left sphenoid sinusotomy, and right medial orbital decompression	Blackish crusts in nasal cavity, inferior turbinate necrosed, maxillary antrum-full of fungal debris, nasolacrimal duct inflamed, pus discharge present	Stable and on treatment
3	Right pansinusitis (M/F/E/S) with right orbital involvement Findings suggestive of invasive fungal sinusitis(STAGE III)	Broad pauciseptate with acute angle branching hyaline septate hyphae observed	*A. fumigatus* & *R. arrhizus*	H& E and PAS stain negative for fungal hyphae, Angioinvasion +	Injection of Lip. Amphotericin B 5125 mg Cumulative dose	B/L Endoscopic debridement		Stable and on treatment
4	O/E B/L nasal polyps	Broad aseptate and thin hyaline septate hyphae suggestive of *Aspergillus* spp.	*R. arrhizus* & *A. flavus*	Sample not observed	Conservative Amphotericin B injection 1mg/Kg For 2 days	ND		Dead
5	Right M/E/F/S sinusitis with blocked anterior and posterior draining pathways and internal contents, subtle mucosal and bony irregularities	1. Broad aseptate perpendicular branching ribbon-like hyphae suggestive of mucormycosis with acute angle branching hyaline septate hyphae seen.	*R. arrhizus* and *A. flavus*	Broad aseptate with thin septate hyphae. Presence of angioinvasion, tissue necrosis, inflammatory cells and giant cell reaction	Amphotericin B injection (1675 mg cumulative dose)	Right total maxillectomy +right orbital exenteration	Blackish crust on middle turbinate and in anterior and posterior ethmoids, pus from the maxilla	Stable and on treatment
6	CXR- B/L Lung opacities; MRI BRAIN-Ethmodal and frontal sinusitis with left orbital cellulitis and optic neuritis. T2/FLAIR hyperintense signal in left anterior temporal lobe-likely Focal Cerebritis	Broad aseptate perpendicular branching with Acute angle branching hyaline septate hyphae seen	*R. arrhizus* and *A. fumigatus*	Broad aseptate along with a few thin septate hyphae. Presence of angioinvasion, tissue necrosis, and inflammatory cells	Liposomal Amphotericin B injection (8100 mg, cumulative dose)	Left total Maxillectomy +leftethmoidectomy+left sphenoid exploration	Necrosis of left maxilla left subcutaneous tissue, left periorbital fat, left inferior recti muscle, left sphenoid sinus mucosa	Stable and on treatment
7	Pansinusitis, findings suggestive of invasive fungal sinusitis with left orbital involvement and perineuritis on left side as described	Broad pauciseptate perpendicular branching ribbon like hyaline hyphae, Acute angle thin hyaline septate	*R. arrhizus* and *A. flavus*	angioinvasion and eosinophils present No fungal elements visible	Amphotericin B injection 1625 mg Cumulative dose	Surgical debridement, FESS with orbital decompression	Polypoid mucosa in right maxillary sinus, anterior and posterior ethmoid and sphenoid Left middle turbinate necrosed, polypodalmucoa in left maxillary sinus, anterior and posterior ethmoid, sphenoid snus	Stable and on treatment
8	Pansinusitis (S/M/E/F), preseptal cellulitis, orbital involvement, optic neuritis, Right eye proptosis	Broad aseptate perpendicular branching ribbon-like hyphae suggestive of Mucormycosis with Acute angle branching hyaline septate hyphae seen.	*R. arrhizus* & *A. flavus*	Broad aseptate along with a few thin septate hyphae. Presence of angioinvasion, tissue necrosis, and neutrophils	Amphotericin B injection (1525 mg cumulative dose)	B/L endoscopic debridement + right orbital exenteration	Lamina papyracea necrosed, infraorbital fat necrosed, blackened tissue present.	Stable and on treatment
9	Right maxillary and b/l ant and posterior sinusitis Findings suggestive of invasive fungal sinusitis	Broad pauciseptate perpendicular branching with acute angle septate hyaline hyphae	*R. arrhizus* & *A. fumigatus*	Broad aseptate along with a few thin septate hyphae. Presence of angioinvasion, tissue necrosis, and neutrophils	Amphotericin B injection (3075 mg, cumulative dose)	B/L endoscopic debridement	Black crust present in B/L anterior and posterior ethmoidal cells and pus in the right maxilla	Stable & on treatment
10	CXR- Non-homogenous opacities in B/L lung CORADS -5, CTSS -38/40 corresponding to 23/25 CT PNS- Soft tissue edema in both nose, Left maxillary sinus polyp	Broad aseptate perpendicular branching ribbon-like hyphae seen with acute angle branching thin hyaline septate hyphae observed.	*R. arrhizus* & *A. flavus*	Sample not sent	Conservative	ND		Dead

**Table 3:** Laboratory findings of 10 COVID-19-associated mucormycosis- aspergillosis patients

	WBC (per mm^3^)		Neutrophil (%)		Haemoglobin (g/dL)		Platelet (per mm^3^)		HbA1C (%)	Procalcitonin (ng/mL)	Serum Ferritin (ng/mL)	S. Creatinine (mg/dL)
	D1	D3	D1	D3	D1	D3	D1	D3				
1	34600	19320	93	94	18.63	12.7	381	146	8.7	3.77	6986.67	4.08
2	7320	10230	51.8	57	11.64	11.64	331.2	421	11.7	0.07	NA	0.92
3	10580	NA	89	NA	12.8	NA	110	NA	9.8	NA	252	0.88
4	22300	18290	95	96	12.17	10	260	183	9.3	11.94	NA	1.2
5	12290	NA	70	NA	10.07	BA	496	NA	9.4	0.08	22	0.55
6	6680	5140	64	63	9.5	8.05	100	100	5.8	0.89	835	4.4
7	21090	14840	91	86	11.67	9.9	207	190	13.4	0.05	NA	0.75
8	12240	10340	86	83	12	10.58	243	269	9.4	0.11	NA	0.95
9	20100	14870	89	84	12.1	10.7	586	374	15.1	NA	402	0.81
10	1462	835	15	31	12.14	7.15	17	70	6.3	3.78	1782	0.88

## Discussion

During the first wave of COVID-19, the primary focus was on CAPA, other fungal superinfections, including *Candida* infections and rare mold infections, whereas the second wave of COVID-19 pandemic, especially in India, was associated with an exponential increase of CAM cases [ 6 , 11 - 13 ]. Rapid diagnosis of secondary infections and co-infections is crucial for the early management of patients. Mucormycosis is a life-threatening entity; however, the true impact of *Aspergillus* co-infections has remained unknown. There is a paucity of data regarding mixed mucormycosis and aspergillosis co-infection in the available literature with no such data available on COVID patients (i.e. cases with CAMA).

Mucorales and *Aspergillus* are ubiquitous soil-dwelling organisms, and humans are exposed daily to fungal elements that dwell in decaying organic matter, soil, animal excreta, dust, compost, foods, spices, unfiltered air, and ventilation system. *Aspergillus* spp. is one of the most commonly isolated fungi among healthy volunteers and is part of their mycobiota [ 14 ]. 

The most important risk factors for mucormycosis and aspergillosis include uncontrolled diabetes mellitus, use of corticosteroids leading to hyperglycemia, extensive use of broad-spectrum antibiotics, prolonged hospitalization, and presence of such co-morbidities as structural lung defects, with diabetes mellitus as the most common risk factor for mucormycosis [ 15 ]. Apart from the aforementioned risk factors, cytokine storm activated by the viral antigens, high airway pressure, lung injury due to long term use of mechanical ventilation, and drug toxicity increases the risk of invasive aspergillosis. Moreover, in ideal situations, immunocompromised patients should be kept in isolation rooms with positive pressure. However, it was recommended to keep COVID-19 positive patients in negative pressure rooms to protect healthcare workers from COVID-19 infection. Ichai et al. also demonstrated that patients admitted in negative pressure ICU rooms were at higher risk of aspergillosis as well other secondary infections [ 16 ].

Patients’ co-morbidities further influence the likelihood of secondary fungal infections [ 17 ]. In this study, patients No. 1, 6, and 7 had a history of cigarette smoking, which is strongly associated with adverse outcomes of COVID-19 in studies [ 18 ]. 

In this study, all patients suffered from COVID-associated pneumonitis either at admission or within the past one month. This suggests that residual pathology from an initial respiratory viral infection can directly damage airway epithelium and facilitate invasion of tissues by molds, such as Mucorales and *Aspergillus* spp. [ 19 ]. SARS CoV-2 causes immune dysfunction or dysregulation leading to a decreased T-cell population which mostly affects the CD4+ CD8+ T-cell subset, induces lymphocytopenia, and eventually results in bacterial and fungal superinfection [ 20 ]. In the case of hematological malignancies, severe lymphopenia has earlier been established as a predicting risk factor for invasive mold disease as observed in patient No. 10 with pancytopenia in this study [ 21 ]. 

Although the triad of COVID pneumonia, diabetes, and steroid use is common for patients worldwide, secondary fungal infections have been exponentially reported from India. This may be attributed to environmental factors, widespread use of steroids, monoclonal antibodies, broad-spectrum antibiotics, antiparasitics, and antivirals as part of cocktail therapy used against COVID-19, even in mild cases. It is worth mentioning that there is no strict prescription check in India for most medicines, including glucocorticoids, which are readily available over the counter. The pre-COVID baseline prevalence of mucormycosis (14 cases per100,000 people) in India is much higher than that in the rest of the world (0.2 cases per 100,000 people) [ 4 ]. In a multicentric study performed in Indian ICUs, *Aspergillus* species were the most common (82.1%) mold isolated [ 22 ]. Moreover, India is the diabetes capital of the world, having the second-largest population with diabetes globally [ 23 ], and the SARS CoV-2 virus has been found to be more prevalent and severe in people with diabetes [ 24 ]. Hyperglycaemia incites hypervirulent strains of specific pathogens and is an independent risk factor for bacterial and fungal co-infections [ 25 ]. 

Irrational use of steroids, either for a long duration or during the early phase of COVID-19 disease, or in higher than recommended doses has been considered the most common underlying causes of fungal superinfection in COVID-19 patients [ 4 ].

**Figure 3:**
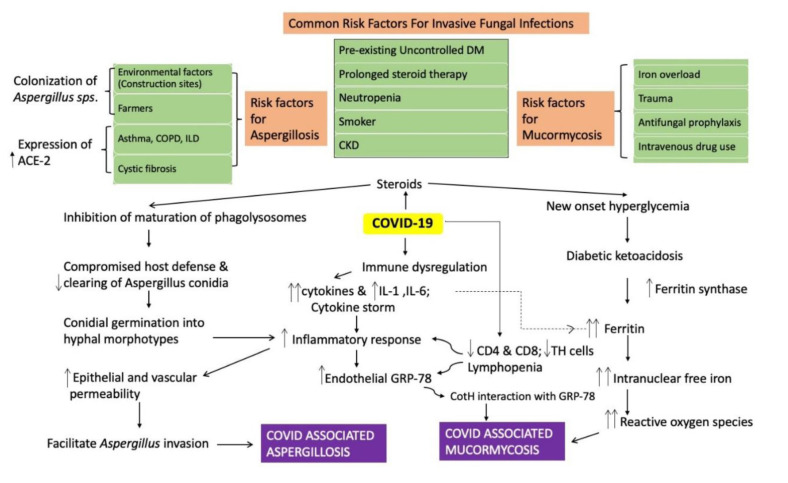
Schematic illustration of the pathophysiological pathway of COVID-19-associated aspergillosis and mucormycosis with associated risk factors

Environmental factors play a significant role in increasing CAM and CAPA in the Indian subcontinent. The atmosphere is more conducive for the fungus to thrive in South Asian developing countries [ 26 ]. In this regard, a few unanswered and unresolved theories refer to the incessant use of industrial-grade oxygen and herbal products as a cause for an increase in fungal diseases. There could also be a possible role of contaminated water used in humidifiers, unsterile humidifier bottles, and non-medical grade industrial oxygen cylinders in increasing fungal infections through the spread of fungal spores [ 27 ]. Even though fungal spores do not survive in waterz high contact hospital surfaces, including reusable humidifier bottles. Considering the above-mentioned viewpoints, the patients in the present study also had such exposures; however, a direct relationship couldn’t be established. A schematic flowchart presenting the pathophy-siological pathway for COVID-19 and associated risk factors for fungal infections is illustrated in Figure 3. 

Patient No. 5 in this study had a history of incision and drainage for dental extraction at a local clinic, suggesting it to be a healthcare-associated case. Few patients in this study had also a history of previous hospitalization, which might have exposed these patients to fungal infections. Studies have reported such secondary fungal infections caused by adhesive bandages, wooden tongue depressors, negative pressure rooms, poor air filtration, non-sterile medical devices, water leaks, and building construction [ 28 ]. Diagnosis of fungal infections is often challenging due to their ubiquitous nature. Direct microscopy (10% KOH mount) and fungal cultures, despite low sensitivity (<50%), remain essential tools in diagnosing fungal infections. Positive culture growth may often reflect laboratory contamination or colonization of nasal tissue and upper respiratory samples, rather than the true disease. Although histopathological examination may confirm invasiveness, the diagnosis is delayed due to prolonged time required for tissue processing and reporting. Further studies must be focussed on the identification of biomarkers to indicate tissue invasion stages and airway involvement for its early diagnosis. Lately, new molecular platforms are being investigated. Mucorales PCR in combination with screening assays for *Aspergillus*, such as serum galactomannan (GM) and (1→3)-β-d-glucan antigen assays, are useful and should be implemented in high-risk cases [ 29 ]. Clinical and radiological findings also need to be correlated with culture findings [ 8 ]. Culture sensitivity has little value in dual infections except for identification of prescence of any other pathogen which has to be manged by non-amphotericin antifungal. In addition, culture details would be essential to reduce amphotericin-B resistance phenomenon in future since no other broad-spectrum drug exists at present.

Regarding the limitations of this study, one can refer to the lack of serum GM test which could have helped identify our cases as proven invasive aspergillosis. Moreover, the obtained fungal isolates were identified only by morphological characteristics, and molecular techniques were not available at our center for the accurate identification of fungal genera at the species level. Despite an early diagnosis of fungal infection post-admission, most participants in this study could not be started on antifungal immediately, and there was an average gap of 4-5 days in the diagnosis and start of antifungal due to limited supply of amphotericin-B (Figure 1). Similar observations were made in a multicentric study conducted in other parts of India [ 30 ]. A delay of about one week in diagnosis may double 30-day mortality from 35% to 66% [ 3 ]. Treating CAMA is more worrisome as dual etiology have to be handled in complete management. This is because for aspergillosis infection with metastatic potential medical treatment with voriconazole is the mainstay while treatment modality for mucor which is a locally invasive pathogen relies equally on surgical and medical management with amphotericin-B. Patient No. 1 was diagnosed about 10 days after admission and could not be saved, reflecting the need for timely diagnosis and treatment.

## Conclusion

MIFIs like CAMA, may become an emerging disease as post-COVID sequelae. These cases were specifically observed in post-COVID patients with uncontrolled diabetes, on steroids, under cocktail therapy, or living in unhygienic environments. Therefore, judicious and relevant use of various medicines (drug stewardship) along with strict maintenance of personal and environmental hygiene is key to preventing CAMA in the post-COVID era. Patients should be advised to keep a check on their glycaemic index and seek medical advice at the earliest. Despite the increased risk of fatality due to CAMA, active multidisciplinary teamwork may help reduce mortality by timely diagnosis and management. The focus should be on empirical management: hit hard approach with both medical and surgical treatment. The study results suggest that timely and accurate diagnosis of CAMA is a must for effective disease management and improvement of outcomes. Mixed fungal infections may often remain unreported. Therefore, identification of risk factors for such cases and measures to prevent the same may help manage invasive fungal infections in COVID patients and decrease overall morbidity. 

## Acknowledgement

We would like to thank the COVID and MUCOR management team, AIIMS, Rishikesh, India, for managing patients and patient data documentation.

## Authors’ contribution

V.S., A.P., and P.K.P. conceptualized the study and contributed to methodology, data curation, and preparation of the original draft. M.T., A.T., and A.T. conducted data visualization and undertook the required investigation. P.K.P., A.K.S., and S.R. supervised the study and did the critical review. A.T. and M.B. reviewed, edited, and validated the final manuscript. All authors approved the final draft of the study.

## Conflict of Interest

None.

## Financial disclosure

The authors declare no financial disclosure. This case series is part of a project entitled disease profile of COVID-l9 (DPC-I9), including patients’ follow-up at a tertiary institute in India, and was approved by the Institutional Ethical Committee in AIIMS, Rishikesh, India.
